# Are We Saving at the Spigot and Wasting at the Bung?

**Published:** 1916-03

**Authors:** 


### Are We Saving at the Spigot and Wasting at the Bung?

The great reduction in medical colleges, the disappearance of all of a sectarian nature except a few high grade homoeopathic institutions, the diminution in medical students and graduates, the higher quality of preliminary and professional education are accomplished facts. But, Christian Science, osteopathy, optometry, cheiropractic and various other cults are widely practiced, are seeking and gradually gaining an official status. Some are pessimistic enough to contend that nothing has really been gained except in mutual esteem within the medical profession, and that the same demand for easy, cheap and low grade medical degrees, with special brands or without them, exists today as heretofore, and that it is being satisfied in markets no longer under the nominal control of the medical profession. We do not entirely agree with this pessimism. It is a considerable advantage to have oleomargarine sold under its own name and not as butter, even if just as much is marketed, and it is a distinct gain that low grade and imitation Medical Doctorates must be sold under another name. But let us not be blind to the fact that the underworld of medicine still exists and thrives in spite of segregation.

